# PARP inhibition in leukocytes diminishes inflammation via effects on integrins/cytoskeleton and protects the blood-brain barrier 

**DOI:** 10.1186/s12974-016-0729-x

**Published:** 2016-09-27

**Authors:** Slava Rom, Viviana Zuluaga-Ramirez, Nancy L. Reichenbach, Holly Dykstra, Sachin Gajghate, Pal Pacher, Yuri Persidsky

**Affiliations:** 1 Department of Pathology and Laboratory Medicine, Temple University, Philadelphia, PA 19140 USA; 2 Center for Substance Abuse Research, Lewis Katz School of Medicine, Temple University, Philadelphia, PA 19140 USA; 3 Laboratory of Cardiovascular Physiology and Tissue Injury, National Institutes of Health/NIAAA, Bethesda, MD 20852 USA

**Keywords:** Leukocyte-endothelial interaction, VLA-4, LFA-1, Actin cytoskeleton, PARP-1

## Abstract

**Background:**

Blood-brain barrier (BBB) dysfunction/disruption followed by leukocyte infiltration into the brain causes neuroinflammation and contributes to morbidity in multiple sclerosis, encephalitis, traumatic brain injury, and stroke. The identification of pathways that decreases the inflammatory potential of leukocytes would prevent such injury. Poly(ADP-ribose) polymerase 1 (PARP) controls various genes via its interaction with myriad transcription factors. Selective PARP inhibitors have appeared lately as potent anti-inflammatory tools. Their effects are outside the recognized PARP functions in DNA repair and transcriptional regulation. In this study, we explored the idea that selective inhibition of PARP in leukocytes would diminish their engagement of the brain endothelium.

**Methods:**

Cerebral vascular changes and leukocyte-endothelium interactions were surveyed by intravital videomicroscopy utilizing a novel in vivo model of localized aseptic meningitis when TNFα was introduced intracerebrally in wild-type (PARP^+/+^) and PARP-deficient (PARP^−/−^) mice. The effects of selective PARP inhibition on primary human monocytes ability to adhere to or migrate across the BBB were also tested in vitro, employing primary human brain microvascular endothelial cells (BMVEC) as an in vitro model of the BBB.

**Results:**

PARP suppression in monocytes diminished their adhesion to and migration across BBB in vitro models and prevented barrier injury. In monocytes, PARP inactivation decreased conformational activation of integrins that plays a key role in their tissue infiltration. Such changes were mediated by suppression of activation of small Rho GTPases and cytoskeletal rearrangements in monocytes. In vitro observations were confirmed in vivo showing diminished leukocyte-endothelial interaction after selective PARP suppression in leukocytes accompanied by BBB protection. PARP knockout animals demonstrated a substantial diminution of inflammatory responses in brain microvasculature and a decrease in BBB permeability.

**Conclusions:**

These results suggest PARP inhibition in leukocytes as a novel approach to BBB protection in the setting of endothelial dysfunction caused by inflammation-induced leukocyte engagement.

**Electronic supplementary material:**

The online version of this article (doi:10.1186/s12974-016-0729-x) contains supplementary material, which is available to authorized users.

## Background

Leukocyte adhesion and migration are mediated by integrins, adhesion receptors expressed on diverse cell types participating in cell-to-cell interactions [1–4]. Integrins facilitate bi-directional signaling. Outside-in transmission involves integrin ligation resulting in stimulation of several signaling pathways. Ligation of other receptors can lead to transmission of an inside-out signal towards the integrin [5]. This signaling usually results in integrin’s conformational modifications (bending/unbending) leading to a rapid rise or decline in integrin-ligand binding affinity and changes in integrin lateral mobility, which directly regulate cell adhesion potential [4, 6] and is critical for immune system responses [6]. Most circulating leukocytes display a non-adhesive phenotype, having integrins in a resting/inactive state. Leukocytes may roll on endothelial cells, arrest, firmly adhere, and transmigrate across the endothelial barrier. In this study, we focused on two major leukocyte integrins [CD49d/CD29, very late antigen-4 (VLA-4), alpha4 beta1 integrin], and [CD11a/CD18, lymphocyte function-associated antigen-1 (LFA-1), alpha L beta2 integrin]. VLA-4 and LFA-1 directly mediate cell arrest under flow conditions, where firm adhesion is mediated by activated (high-affinity, unbent) integrins. The activation of integrins, adhesion and migration processes involve a chain of actin cytoskeleton rearrangements and activation of small Rho GTPases [6–9].

In the search for anti-inflammatory/neuroprotective compounds, we focused on poly(ADP-ribose) polymerase-1 (PARP) inhibitors (further referred to as PARPi) that are currently used in the treatment of cancer [10]. PARP is responsible for synthesis and transfer of ADP-ribose polymers to target proteins, regulation of DNA repair, and genomic integrity maintenance [11]. PARP hyperactivity has been found in a number of central nervous system (CNS) disorders (including ischemia, neurodegeneration, and neuroinflammation) [12]. PARP inhibition in macrophages/microglia diminished production of inflammatory mediators [11], decreased the number of T cell subsets, while increasing the amount of anti-inflammatory cytokines and regulatory, suppressive T lymphocytes [13]. The first PARPi were discovered in 1980 [14, 15]. All known PARPis are nicotinamide mimetics that bind in the NAD+ binding pocket of the catalytic domain, with the exception of indole and coumarin derivatives that bind in the zinc-finger domains in PARP [14–16]. In the current study, we used PARPis belonging to the nicotinamide mimetic group; two of them (olaparib and talazoparib) are currently in clinical trials for treatment of several malignancies (phase II and III), offering rapid translation to therapy of immune/inflammatory disorders [10]. PARPi have been shown to be anti-inflammatory and tissue-protective in animal models of traumatic brain injury (TBI) [17], multiple sclerosis (MS) [18], meningitis [19], arthritis [20], and lung, liver, and kidney injury [21].

Inflammation plays a significant role in neuronal dysfunction and neurocognitive impairment [22]. Neuroinflammatory responses are characterized by activation of resident macrophages and microglia that release cytokines, chemokines, and proteolytic enzymes negatively affecting neural cells. Inflammatory molecules can stimulate the brain endothelium from the abluminal (brain) side and increase expression of adhesion molecules on the luminal side [23], leading to enhanced leukocyte engagement of the brain endothelium and subsequent migration across the blood-brain barrier (BBB) into the neuropil, further aggravating neuroinflammation [24]. In systemic inflammation (e.g., sepsis) [25], there is an enhanced adhesion of leukocytes in different organs (including CNS) as well as cytokine production that result in increased in BBB permeability. During stroke, in response tissue injury/local cytokine/chemokine production, neutrophils infiltrate ischemic brain (in 30 min to a few hours), while monocytes/macrophages migrate within a day or two, and T cells follow on day 3 to 7 after an ischemic event [26, 27]. Lymphocyte/monocyte infiltration into the CNS is a signature event in multiple sclerosis (MS), an inflammatory condition of the CNS. It remains enigmatic whether BBB dysfunction leads to immune cell infiltration or it is the result of perivascular leukocyte accumulation, but leukocyte migration definitely modifies BBB permeability. Leukocytes in MS express inflammatory cytokines, reactive oxygen species (ROS), and enzymes that can augment their egress into the CNS by influencing BBB function, either directly or indirectly [28, 29]. This process is accompanied by BBB injury and increased permeability to blood components toxic to neurons [30]. Therefore, approaches diminishing leukocyte interactions with brain endothelial cells may prevent BBB damage and attenuate neuroinflammation, resulting in neuroprotection [31]. Inhibiting leukocyte migration during the first stages of inflammation (in sepsis, MS, or ischemia/reperfusion) may have positive effects by reducing tissue injury, but on the other hand, prolonged inhibition might have negative effects in terms of uncontrolled infections or prevention of immune cells from initiation of regenerative activities, since inflammation is a vital part of the physiological reaction towards tissue repair and plasticity [32–34]. It is essential to look for anti-inflammatory treatments and to study their mechanisms of action to be able to fine tune therapeutic approaches.

Our prior work revealed that PARP inhibition in primary human brain microvascular endothelial cells (BMVEC) decreased monocyte adhesion and migration across BMVEC monolayers and attenuated expression of adhesion molecules. PARP suppression in BMVEC attenuated activity of small GTPases, dampened expression and secretion of pro-inflammatory factors [35]. Utilizing an in vivo aseptic meningitis/encephalitis model, we demonstrated diminished adhesion and migration of leukocytes across the BBB, confirming our in vitro observations.

While we could selectively inhibit PARP in vitro in BMVEC, it was impossible to distinguish whether decreased adhesion/migration in vivo resulted from PARP inhibition in endothelium or from PARP suppression in leukocytes as well. To challenge this issue, we used adoptive transfer of leukocytes and intravital videomicroscopy (IVM) and found that leukocytes from PARP1^−/−^ mice (PARPko) displayed reduced adhesion and migration across cortical vessel endothelium vs. their wild-type PARP1^+/+^ (WT) counterparts. To address putative anti-inflammatory effects of PARP inhibition in leukocytes, we pretreated human monocytes with PARPi and showed their decreased adhesion/migration across BBB models, paralleling attenuation of active integrin expression, cytoskeletal alterations, and GTPase activity. Genetic or chemical inhibition of PARP in vivo prevented their migration across the BBB and diminished barrier permeability.

## Methods

### Reagents and cell culture

The selective PARPi used were 5-aminoisoquinolinone (AIQ; Enzo Life Sciences, Farmingdale, NY); olaparib and talazoparib (Selleck Chemicals, Houston, TX), 5′-deoxy-5′-[4-[2-[(2, 3-dihydro-1-oxo-1H-isoindol-4-yl) amino]-2-oxoethyl]-1-piperazinyl]-5′-oxoadenosine dihydrochloride (EB47; Tocris Bioscience, Minneapolis, MN). The RhoA inhibitor, CT04, and RhoA/Rac1 activator, CN04, were from Cytoskeleton (Denver, CO). The Rac1 inhibitor, NSC23766, was from EMD Millipore (Billerica, MA). Lipopolysaccharide (LPS) from *Escherichia coli* 0111:B4 and Rhodamine 6G were from Sigma-Aldrich (St. Louis, MO). Recombinant human tumor necrosis factor-α (TNFα) and human monocyte chemotactic protein type-1 (MCP1/CCL2) were from R&D Systems (Minneapolis, MN). Phorbol 12-myristate 13-acetate (PMA) was from Cayman Chemical (Ann Arbor, MI).

Primary human monocytes from HIV-1/hepatitis B seronegative donors were obtained from the University of Nebraska Medical Center [36]. Primary brain microvascular endothelial cells (BMVEC) were provided by Dr. M. Witte (University of Arizona, Tucson, AZ), isolated from the temporal cortex obtained during surgical removal of eleptogenic foci in adult patients [37] and maintained in culture as described [9]. Cell culture reagents were from Life Technologies (Carlsbad, CA).

Monocytes were pretreated with PARPi (AIQ 1 μM, olaparib, EB47, 10 μM; talazoparib, 10, 25, or 10 nM) [35, 38, 39] or RhoA/Rac1 activator/inhibitor (CN04, 1 μg/ml; CT04, 1 μg/ml; NSC23766, 75 μmol/l) for 30 min unless otherwise noted and did not affect cell viability [39]. Dose-response results are shown in Additional file 1: Figure S1. In all experiments, the designation non-treated (NT) indicates that medium only was added to the cells.

### Animals

C57BL/6 mice (8-week-old male) were from the Jackson Laboratory (Bar Harbor, MI). PARPko mice (PARP1^−/−^) were generated on C57BL/6J background [40] provided by Dr. P. Pacher (NIAAA). To achieve statistical significance in each experiment, mice were divided into groups of four to six animals (exact numbers for each experiment are indicated in figure legends).

All in vivo experiments were approved by the Temple University Institutional Animal Care and Use Committee in accordance with guidelines based on the National Institutes of Health (NIH) guide for care and use of laboratory animals and ARRIVE (Animal Research: Reporting In Vivo Experiments) guidelines (www.nc3rs.org.uk/arrive-guidelines).

### IVM and ex vivo treatment and labeling of leukocytes

Prior to IVM, mice underwent craniotomy and cranial window implantation as described [41]. Prior to IVM, mice were treated with LPS (6 mg/kg) i.v. or TNFα (0.5 μg/mouse) by IC injection [9, 35, 42, 43]. Two hours post-injection of TNFα, leukocytes were labeled in vivo with Vybrant® DiI Cell-Labeling Solution (DiI) (Life Technologies, Carlsbad, CA) introduced i.v. Leukocyte adhesion was detected in cerebral vessels through the cranial window using a SteREO Discovery V20 epifluorescence microscope (Carl Zeiss Microimaging Inc., Thornwood, NY) equipped with a AxioCam MR digital camera as previously described [35, 44]. A 30-s video (time-series image set between 16 and 20 frames/s) was captured using the digital high-speed recorder. Adherent leukocytes were defined as the number of leukocytes firmly attached to the endothelium that did not change location during the observation period, scored as the number of cells per mm^2^ of the vascular surface area, calculated from the diameter and length of the vessel segment under observation. Imaris 8.3 software (Bitplane AG, Zurich, Switzerland) was used to count adherent leukocytes. Transmigrated leukocytes were enumerated 24 h later in an area covering a distance of 100 μm from the pial and parenchymal vessel wall by epifluorescent IVM. The number of extravasated leukocytes was counted and normalized to area, using ImageJ software (National Institutes of Health, Bethesda, MD) [35].

Leukocytes were isolated from five donor mice PBMC with red blood cell lysis buffer (eBioscience, Inc., San Diego, CA). Leukocytes (2 × 10^6^) were ex vivo treated with PARPi for 30 min, washed with PBS, and labeled with calcein-AM (1 μM, Life Technologies) as described [9]. Concomitantly, recipient mice were treated with LPS or TNFα as described above for 4 h, and mice were anesthetized, injected intra-orbitally with Rhodamine 6G to label autologous leukocytes, imaged by IVM, and then injected with calcein-AM-labeled leukocytes. Leukocytes were visualized by fluorescent light (495 or 601 nm excitation for calcein-AM or Rhodamine 6G, respectively). Ex vivo treatment of leukocytes did not change the population profile (Additional file 1: Figure S2B).

### In vivo permeability assay

Changes in BBB permeability were assessed using the fluorescent tracer, sodium-fluorescein (Na-F) as described [35, 41, 43]. Briefly, to prevent blood clotting in the vessels, animals were injected i.p. with heparin (20 U) followed i.v injection of 50 μl of 2 % Na-F in PBS. The tracer was allowed to circulate for 30 min. The mice were anesthetized and then transcardially perfused with PBS until colorless perfusion was visualized. The animals were then decapitated and the brains were quickly isolated. After removal of the meninges, cerebellum, and brain stem, the tissue was weighed and homogenized in 10 times volume of 50 % trichloroacetic acid. The homogenate was centrifuged for 10 min at 13,000×*g*, and the supernatant was neutralized with 5 M NaOH (1:0.8). The amount of Na-F was measured using a Synergy 2 plate reader (BioTek, Winooski, VT). Fluorescent dye content was calculated using external standards; data are expressed as amount of tracer per milligrams of tissue.

### PARP activity assay

To measure PARP activity, leukocytes were treated with/out PARPi for 30 min and then lysed and subjected to ELISA according to manufacturer’s instructions (Trevigen, Gaithersburg, MD).

### Conformational changes of VLA-4 and LFA-1 integrins

To assess VLA-4 conformational status in monocytes, the VLA-4-specific ligand, l-leucyl-l-aspartyl-l-valyl-l-prolyl-l-alanyl-l-alanyl-l-lysine (LDV) (Tocris), was used. The activated conformation of VLA-4 was measured by flow cytometry (FACS) using HUTS21 Ab as described [8]. To assess LFA-1 conformational status, monocytes (0.5 × 10^6^ cells/ml in RPMI medium/1 % FBS) were first treated with/out inhibitors and then stimulated with PMA (100 ng/ml) for 1 h. To prevent RhoA or Rac1 GTPase activity, cells were pretreated with specific inhibitors, 1 μg/ml CT04 (Cytoskeleton Inc.) or 75 μM NSC23766 (EMD Millipore, Billerica, MA), respectively. Monocytes were placed on ice and fixed with 4 % formaldehyde. Activated LFA-1 conformation was detected by FACS with MEM-148 Ab. Data were acquired with a FACS BD Canto II flow cytometer (BD Biosciences, San Jose, CA) and analyzed with FlowJo software (Tree Star, Inc., Ashland, OR). Data were collected from at least 10,000 events for each experimental condition and repeated with monocytes from three different donors. Quantitation of integrin conformational activation was performed, where the mean fluorescence intensity (MFI) of activated non-treated cells was assigned a value of 100 and the value of 0 was assigned to the MFI of cell autofluorescence.

### RhoA and Rac1 guanosine triphosphatase (GTPase) activity assay

RhoA and Rac1 GTPase activities were measured by G-LISA RhoA and Rac1 activation assay kits (Cytoskeleton Inc., Denver, CO) in cell lysates prepared from monocytes (with/out PARPi) after stimulation with MCP-1 (30 ng/ml) or PMA (100 ng/ml) for 1 h. To inhibit RhoA or Rac1 GTPase activity, cells were pretreated with specific inhibitors, 1 μg/ml CT04 (Cytoskeleton Inc.) or 75 μM NSC23766 (EMD Millipore, Billerica, MA), respectively. Monocytes stimulated with CN04 (Cytoskeleton Inc.) served as a positive control for Rho activity.

### Quantification of F-actin and G-actin

Monocytes were treated with PARPi or RhoA/Rac1 inhibitors and stimulated by MCP-1 as described above, washed with ice-cold PBS, and fixed with 4 % formaldehyde. Filamentous actin (F-actin) and globular actin (G-actin) were stained by Acti-stain™ 488 phalloidin (Cytoskeleton) and DNase 1-Alexa 591 (Life Technologies), respectively. Data were collected from at least 20,000 events for each sample by a BD FACS Influx and analyzed as described above. The F/G actin ratio was calculated by dividing the mean fluorescent intensity (MFI) of F-actin by the MFI of G-actin.

### Monocyte adhesion assay

BMVEC monolayers were treated overnight with TNFα (20 ng/ml). Monocytes were treated overnight with/out PARPi and washed prior to calcein-AM labeling. Fluorescence of adherent monocytes was measured using a Synergy 2 plate reader (BioTek, Winooski, VT) as described [8]. Results are presented as the mean ± SEM fold adhesion (number of adherent monocytes for each experimental condition divided by the basal adhesion of the untreated control), which was assigned a value of 1 (7600 relative fluorescent units).

### Transendothelial migration assay

Monocytes were treated with PARPi, labeled as described above, and washed before addition to BMVEC. FluoroBlok (BD Bioscience, Bedford, MA) cell culture inserts, intended to block the transmission of fluorescent light between 490 and 700 nm, were used to permit continuous detection of fluorescently labeled monocytes migrating across endothelial monolayers. BMVEC were seeded on collagen type I coated 3-μm pore 24-well tissue culture FluoroBlok inserts at a density of 2.5 × 10^4^ cells per insert. Confluent monolayers were then exposed to TNFα (20 ng/ml) for 24 h to activate the BMVEC. After activation, BMVEC were rinsed with fresh medium and the medium was replaced. For migration assays, calcein-AM-labeled monocytes were added to the upper chamber of the tissue culture insert system, while the chemoattractant, CCL2/MCP-1 (30 ng/ml), was added to the lower chamber to stimulate migration. Chemotaxis towards MCP-1 was allowed for 2 h as described [8]. The number of migrated monocytes was determined using ImageJ software (NIH) and presented as fold difference in migration from triplicate determinations, calculated from the number of migrated monocytes for each experimental condition divided by the number of migrated monocytes in the untreated, no chemoattractant control (assigned a value of 1, equivalent to 37 migrated cells).

### Transendothelial electrical resistance (TEER)

To determine the integrity of brain endothelial monolayers after engagement with monocytes, TEER was measured using the 1600R ECIS system (Applied Biophysics, Troy, NY). Using the free ions in the culture media, the instrument generates an AC current flow between the electrode and counter electrode located in specialized tissue culture arrays and measures the change in impedance. The ECIS system provides real-time complex impedance, providing readouts for impedance, resistance, and capacitance. In brief, BMVEC were plated on collagen-coated electrode arrays (96W20idf) and maintained until a monolayer formed with a TEER of 800–1500 Ω. Monocytes were treated with/out PARPi overnight, rinsed prior to the addition of monocytes (1 × 10^5^ cells/well) to the BMVEC. Measurements were taken every 30 min at 4000 Hz as described [39, 43]. The results are presented as the percent change from baseline TEER from at least two independent experiments (expressed as average ± SEM) consisting of four to six replicates each (100 % equals a resistance of 644 Ω).

### Statistical analysis

The results are expressed as the mean ± SEM of experiments conducted multiple times. Multiple group comparisons were performed by one-way analysis of variance with Dunnet’s post hoc tests. Statistical analyses were performed utilizing Prism v5 software (GraphPad Software Inc., La Jolla, CA). Differences were considered significant at *P* values <0.05.

## Results

### PARPko mice exhibit decreased leukocyte adhesion to and migration across the brain endothelium in vivo and attenuated enhanced BBB permeability in TNFα-induced encephalitis

**Fig. 1:**
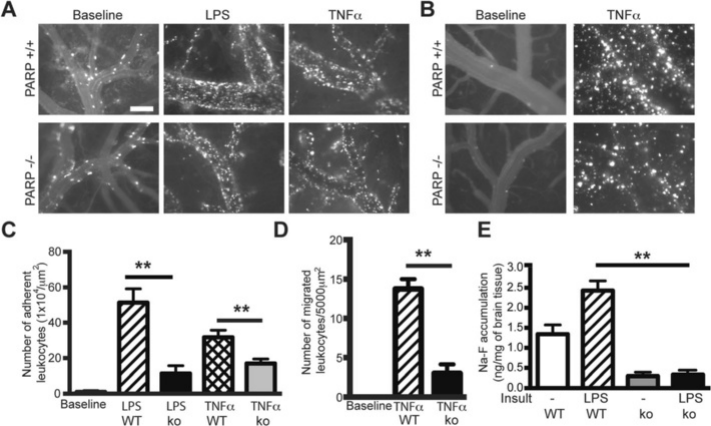
PARP deficiency diminishes leukocyte adhesion to and migration across the BBB and reduces BBB permeability. Representative images from videos of leukocytes labeled with Rhodamine 6G for adhesion (**a**) and migration (**b**). Measurement of labeled leukocytes after their adhesion to (**c**) or migration (**d**) across the endothelium. **e** Measurement of BBB permeability in vivo by sodium-fluorescein accumulation in the brain in the presence or absence of LPS. Results are shown as mean adhesion or migration ± SEM (four animals per treatment group). **P < 0.01 indicates significance vs. TNFα or LPS-treated animals. Scale bar = 100 μm is shown in one micrograph, but it is valid for all micrographs shown

Recently, we demonstrated that PARP inhibition in BMVEC lessened monocyte adhesion and migration across BMVEC monolayers and attenuated expression of adhesion molecules [35]. To further assess effects of PARP inhibition on leukocyte adhesion/migration, we used IVM in PARPko and WT mice, utilizing systemic LPS-induced inflammation [35] and TNFα-mediated encephalitis (by IC injection). In WT mice, LPS and TNFα treatment resulted in 52- and 42-fold increases in leukocyte firm adhesion, respectively, whereas PARPko mice showed decreased level of leukocyte adhesion with both LPS (74 %) and TNFα (25 %) (Fig. 1a, c).

IVM showed increased leukocyte migration across the brain endothelium after IC TNFα administration by 14.5-fold vs. baseline in WT mice, while PARPko mice displayed only a 2.6-fold increase vs. baseline (Fig. 1b, d). Because leukocyte engagement of endothelium and inflammation results in enhanced permeability of the BBB, we determined the effect of PARP inhibition on barrier permeability in a LPS-induced systemic inflammation model. The amount of Na-F in the whole brain was elevated 1.7-fold after LPS-treatment of WT mice compared to untreated WT mice (Fig. 1e) whereas in PARPko mice, Na-F accumulation was significantly decreased in untreated and LPS-treated PARPko animals as compared to WT mice (Fig. 1e). These results further indicate that suppression of PARP activity protects BBB integrity.

### Inhibition of PARP decreases leukocyte adhesion to the brain endothelium in an adoptive transfer mouse model of LPS-induced systemic inflammation

**Fig. 2:**
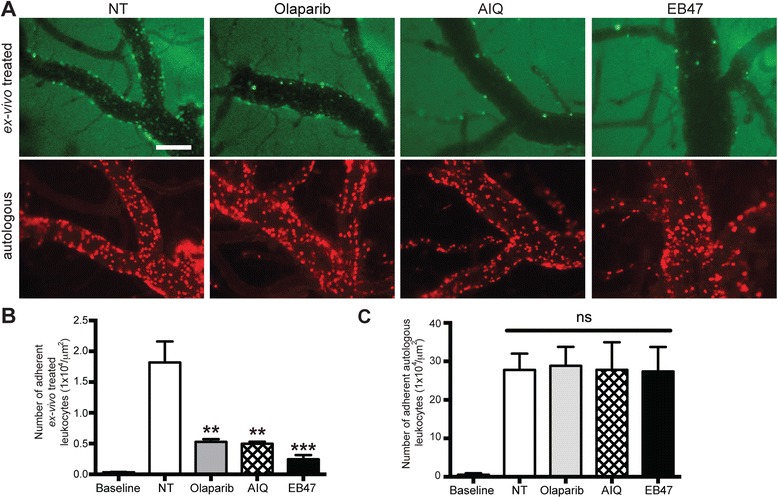
PARP inhibition decreases leukocyte adhesion in brain microvessels during systemic inflammation. **a** Representative images from videos of leukocytes labeled with calcein-AM. **b** Measurements of leukocyte firm (not rolling) adhesion during a 30-s observation period (ex vivo treated, calcein-AM-labeled leukocytes). Leukocytes were treated ex vivo with or without PARP inhibitors for 4 h. **c** Measurements of leukocyte firm (not rolling) adhesion during a 30-s observation period (autologous, Rhodamine 6G-labeled leukocytes). Results are shown as mean adhesion ± SEM (four to six animals per treatment group). *ns* indicates no significance, **P < 0.01, ***P < 0.001 indicates significance vs. NT. Scale bar = 100 μm is shown in one micrograph, but it is valid for all micrographs shown

Our previous work utilizing systemic PARPi administration [35] or PARPko animals (Fig. 1) did not allow us to determine whether the effects observed were due to diminution of inflammatory responses in endothelial cells or in leukocytes. Therefore, we evaluated the effect of selective PARP inhibition in leukocytes on their adhesion in vivo utilizing adoptive transfer. Leukocytes were isolated from WT mice, treated ex vivo with/out PARPi, labeled with calcein-AM, washed, injected into LPS-treated WT mice, and monitored by IVM. Olaparib and EB47 ex vivo treatment reduced PARP activity to levels close to PARPko leukocytes, whereas AIQ reduced PARP activity by 53 % (Additional file 1: Figure S2A). LPS treatment increased leukocyte attachment (adhesion) >40-fold to the brain endothelium, while leukocytes ex vivo treated with PARPi displayed significantly decreased adhesion compared to untreated leukocytes by 71–87 % (Fig. 2a, b). Mice injected with LPS showed ~30-fold increase in firm adhesion of autologous (non-treated) leukocytes to the cortical endothelium (Fig. 2c). These results demonstrate that PARPi have potent in vivo anti-inflammatory properties in leukocytes.

### PARP inactivation in leukocytes attenuates adhesion to the brain endothelium

**Fig. 3:**
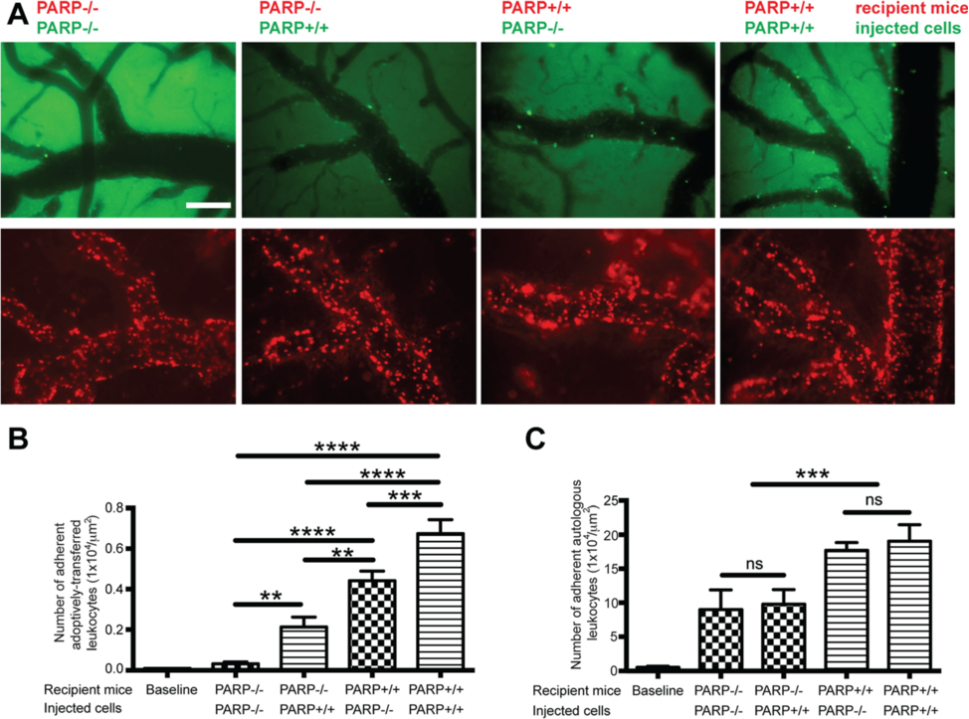
PARP ko mice show diminution of leukocyte adhesion to the brain endothelium in vivo. **a** Representative images from videos of leukocytes labeled with calcein-AM (*upper panels*) or Rhodamine 6G (*lower panels*). **b** Measurement of labeled leukocytes under firm (not rolling) adhesion in WT or PARP ko mice during a 30-s observation period (ex vivo treated, calcein-AM-labeled leukocytes). **c** Measurements of leukocyte firm (not rolling) adhesion during a 30-s observation period (autologous, Rhodamine-labeled leukocytes). Results are shown as mean adhesion ± SEM (four animals per treatment group). *ns* indicates no significance, **P < 0.01, ***P < 0.001, ****P < 0.0001 indicates significance vs. LPS treated. Scale bar = 100 μm is shown in one micrograph, but it is valid for all micrographs shown

Since administration of PARPi significantly reduced leukocyte adhesion to cortical vessels, we hypothesized that PARP inhibition would affect the ability of leukocytes to adhere to cortical vessels. We performed adoptive transfer where ex vivo-labeled leukocytes, isolated from PARPko animals, were i.v.-injected into recipient WT animals and vice versa, utilizing a LPS-induced systemic encephalitis model. PARPko leukocytes transferred to WT mice exhibited 36 % decreased adhesion compared to WT PARP-expressing leukocytes injected to WT mice (Fig. 3a, b). When PARPko leukocytes were transferred to PARPko mice, leukocytes showed even further decrease in adhesion. Whereas the cells from the PARPko mice were able to adhere much better in the WT mice compared to the PARPko mice mimicking results in Fig. 1c, and leukocyte adhesion in the PARPko animals was reduced too. Adhesion of autologous leukocytes did not change, regardless of the type of transferred leukocytes (Fig. 3c). These results support our idea that inhibition of PARP in leukocytes affects their capability to adhere to endothelium.

### PARP suppression in monocytes decreases adhesion to BMVEC monolayers and transendothelial migration

**Fig. 4:**
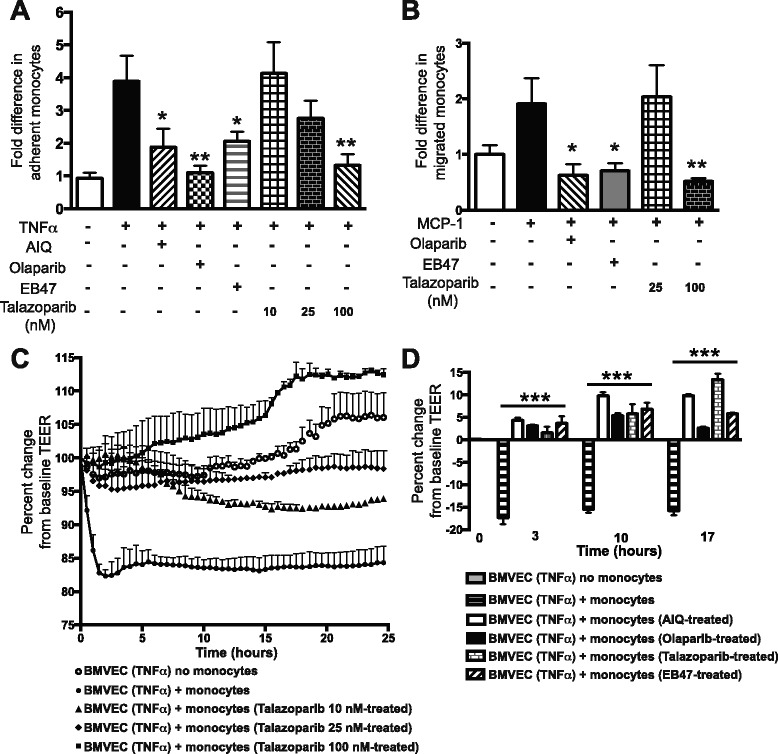
PARP inhibition prevents adhesion to and migration of monocytes across BMVEC monolayers preserving the barrier. Primary human monocytes were treated for 24 h with PARPi (AIQ, olaparib, EB47, talazoparib), calcein-labeled, washed, and then added to BMVEC monolayers (untreated or treated for 24 h with TNFα). Treatments were removed prior to the addition of monocytes. Adhesion to (**a**) and migration of (**b**) monocytes across blood-brain barrier models were measured and are presented as fold difference compared to TNFα-only control (mean ± SEM) for each treatment from at least quadruplicate determinations, which was assigned a value of 1 (7600 relative fluorescent units for adhesion or equivalent to 37 migrated cells). *P < 0.05, **P < 0.01 indicate significance vs. non-treated. TEER, an indicator of barrier integrity, was continuously measured in BMVEC monolayers treated with or without TNFα following the addition of primary human monocytes that had been treated with PARPi. **c** Dose-dependent treatment of monocytes with talazoparib at 10, 25, or 100 nM. **d** Treatment of monocytes with AIQ, olaparib, EB47 at 10 μM, and talazoparib at 100 nM; the percent change in TEER at 3, 10, and 17 h is shown. Data are presented as the percent change from baseline TEER (mean ± SEM) for at least six replicates (100 % equals a resistance of 644 Ω). ***P < 0.005 indicate significance vs. non-PARPi treated

We determined whether our in vivo observations could be confirmed in an in vitro BBB system utilizing primary human monocytes and human BMVEC. Monocytes were treated with PARPi, washed and added to TNFα-stimulated BMVEC. Monocyte adhesion to BMVEC was increased nearly fourfold following TNFα treatment and was significantly inhibited by the PARPi, AIQ, Olaparib, and EB47 (by 55, 91, and 47 %, respectively) (Fig. 4a). In dose-response experiments, talazoparib reached a significant (86 %) decrease in adhesion at 100 nM. We next determined if PARP inhibition in monocytes would affect their transendothelial migration through BMVEC monolayers. Monocytes were treated with PARPi; after treatments were removed, MCP-1 was added to the lower chamber of BBB constructs, which resulted in a twofold increase in monocyte migration towards the chemoattractant. Monocyte migration was completely abolished by PARPi, olaparib, EB47, and talazoparib (Fig. 4b). These in vitro protective effects observed with PARPi on monocyte/endothelial interactions in adhesion/migration assays are consistent with our in vivo results.

### PARP inhibition prevents monocyte-mediated disruption of the BBB

To understand if PARP inhibition in monocytes could diminish their activation, lessen BMVEC engagement, and preserve barrier integrity in brain endothelial cells in vitro, we used TEER to assess barrier tightness in BMVEC monolayers. BMVEC demonstrated the expected steady state barrier formation, but monocyte addition resulted in a rapid diminution (15–20 %) in resistance (mimicking BBB injury in vivo) that was maintained for the experiment’s duration. Dose-response monocyte exposure to talazoparib, significantly attenuated monocyte engagement-induced drop in resistance, enhancing TEER by 7–10, 10–13, and 17–27 % (Fig. 4c). AIQ, olaparib, and EB47 also prevented barrier injury, as demonstrated by 3–10 % increases in TEER (Fig. 4d). Taken together, these results demonstrate that PARP inhibition in monocytes decreases their engagement with endothelium and protects barrier integrity in vitro.

### Inhibition of PARP prevents conformational activation of VLA-4 and LFA-1 integrins in monocytes

**Fig. 5:**
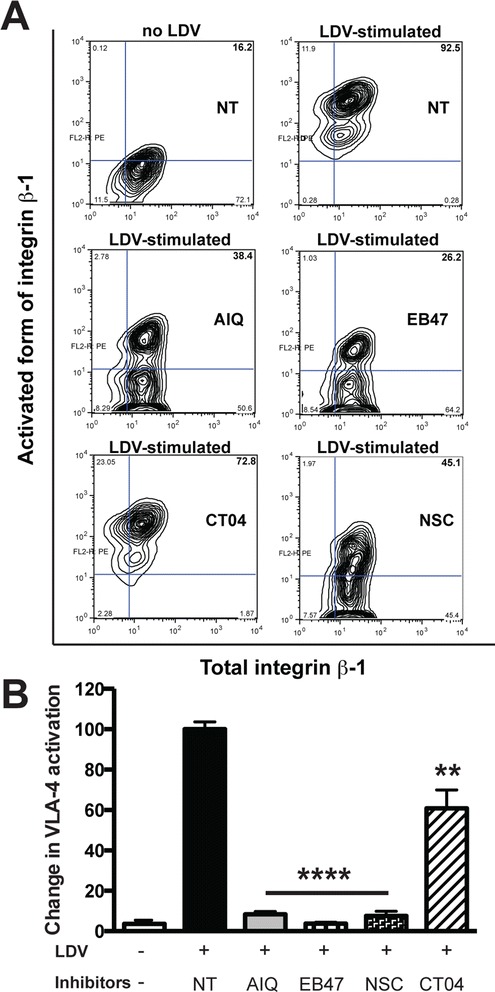
PARP inhibition prevents conformational activation of VLA-4 (integrin β1) in monocytes. Monocytes were treated with PARPi (AIQ or EB47) or RhoA or Rac1 GTPase inhibitor (CT04 or NSC23766). The expression of total VLA-4 (CD29) was unaffected by treatment with PARP inhibitors. **a** The active form of VLA-4 was detected using HUTS21, a conformation-specific antibody. Integrin conformation in monocytes changes from a closed (inactive) to an open (active) form after LDV stimulation. Total amount of integrin β1 (CD29) is shown on the *x*-axis. **b** Quantitation of integrin β1 conformational activation, expressed as the percent of monocytes containing the activated integrin conformation (as shown in the right upper quadrant of each graph in panel **a**). Results are presented as the mean ± SEM (**P < 0.01, ****P < 0.005 vs. untreated control) from three independent experiments

The adhesion of monocytes to activated endothelial cells is governed by conformational changes in the integrins, including VLA-4 and LFA-1 [8]. We evaluated expression of active forms of VLA-4 following stimulation with LDV (a peptide mimicking activation during exposure to the adhesion molecule, VCAM-1, and fibronectin), which resulted in a change in integrin β1 conformation from inactive (closed/non-adherent) to active (open/adherent) form. PARPi treatment of monocytes drastically decreased LDV-induced expression of active integrin β1, as did treatment with Rac1 inhibitor, NSC23766 (>90 %) (Fig. 5). The RhoA inhibitor, CT04, decreased this to a lesser degree (40 %).

**Fig. 6:**
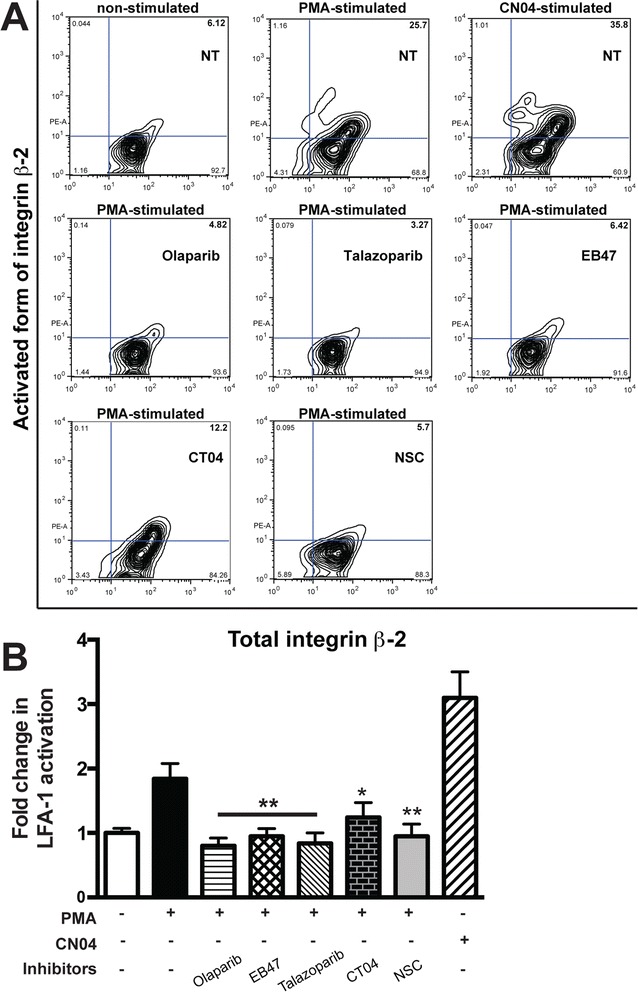
PARP suppression diminishes conformational activation of LFA-1 (integrin β2) in monocytes. Monocytes were treated with PARPi (olaparib, EB37, or talazoparib) or RhoA and Rac1 GTPase inhibitor (CT04 or NSC23766). Expression of total LFA-1 (CD18) was not affected by treatment with PARPi. The activated form of LFA-1 was detected using MEM-148, a conformation-specific antibody. PMA-stimulation of monocytes changes integrin conformation from a closed (inactive) to an open (active) form (**a**). The total amount of integrin β2 (CD18) is shown on the *x*-axis. **b** Quantitation of integrin β2 conformational activation, expressed as the percent of monocytes containing the activated integrin conformation (as shown in the right upper quadrant of each graph in panel **a**). Results are presented as the mean ± SEM (*P < 0.5, **P < 0.05 vs. untreated control) from three independent experiments

Activation of primary human monocytes by PMA resulted in a significant twofold upregulation of the active form of LFA-1, while exposure to olaparib, EB47, or talazoparib resulted in a significant decline of activated LFA-1 compared to untreated monocytes (Fig. 6). Rac1 inhibitor treatment also significantly decreased LFA-1 activation (*P* < 0.05). However, RhoA inhibitor treatment reduced LFA-1 activation with lesser extent (*P* < 0.5).

### PARP inactivation in monocytes affects actin cytoskeleton

**Fig. 7:**
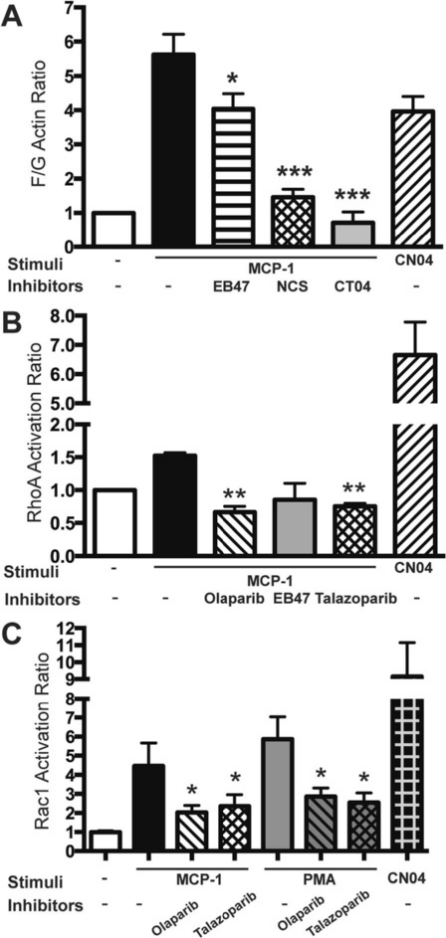
PARP inhibition suppresses the activation of GTPases in monocytes and decreases cytoskeletal changes. Monocytes were pretreated with PARPi (olaparib or talazoparib) or RhoA or Rac1 inhibitor (CT04, NSC23766) before stimulation by MCP-1. **a** F/G actin ratio in monocytes treated with PARP inhibitors or RhoA/Rac1 inhibitors. G-LISA was used to measure RhoA (**b**) and Rac1 GTPase activity (**c**) in cell lysates prepared from the monocytes. The level of RhoA or Rac1 activity in non-stimulated, non-treated cells was assigned a value of 1. Results are shown as the mean ± SEM (*P < 0.5, **P < 0.05 vs. untreated control) from three independent experiments

We next measured PARP inhibition effects on actin cytoskeleton rearrangements in monocytes. MCP-1-stimulation of monocytes resulted in a 5.6-fold increase in F/G actin ratio (Fig. 7a). PARPi treatment of monocytes resulted in a 28 % decrease in F/G action ratio. Specific RhoA and Rac1 inhibitors also decreased F/G actin ratio, indicating that PARP inhibition affects the actin cytoskeleton via RhoA and Rac1 inhibition. We next evaluated the activation of the small GTPases, RhoA, and Rac1, in monocytes, in view of their role in the actin cytoskeleton rearrangements during their adhesion and migration. MCP-1-stimulated monocytes showed increased RhoA by 1.6 (Fig. 7b). MCP-1- or PMA-stimulated monocytes exhibited amplified Rac1 activation by 4- and 5.9-fold, respectively (Fig. 7c). PARPi, olaparib, and talazoparib significantly reduced activation of both Rho GTPases.

## Discussion

Leukocyte trafficking from blood into tissues is a vital response to injury and involves multiple steps mediated by adhesion molecules and chemoattractants. Inflammation and its associated infiltration of various leukocyte types, as well as leukocyte-endothelium interaction, play a significant role in numerous CNS diseases; therefore, the molecular mechanisms controlling leukocyte migration are potential therapeutic targets. Inflammatory and immune cells play complex roles after ischemic stroke, MS, encephalitis, and meningitis [10, 19, 45–48]. Actin cytoskeleton machinery, integrins, and their conformational changes are key elements in the ability of leukocyte subsets to roll on endothelial cells, arrest and firmly adhere, and transmigrate across the BBB, leading to its dysfunction and neuroinflammation resulting in neuronal dysfunction. Lessening leukocyte adhesion and migration might serve as an ultimate tool in weakening of inflammatory events in the brain.

PARP suppression has been shown to diminish edema, preserve the tight junction protein, occludin, and decrease expression of the adhesion molecule, ICAM-1, in animal models of stroke, traumatic brain injury, meningitis, and MS [45, 49–51], reducing leukocyte infiltration and brain inflammation [49, 52]. Application of PARPi has been previously shown to reduce leukocyte adhesion [49, 52]. However, these studies did not address whether the effects of PARP inactivation apply to immune cells, to endothelium or to both. Recently, we showed that PARP inhibition caused a significant reduction in leukocyte adhesion/migration in a novel in vivo model of localized neuroinflammation as well as in systemic inflammation, thereby preserving BBB integrity [35]. Additionally, inhibition of PARP in BMVEC resulted in attenuation of expression of adhesion molecules, such VCAM-1 and ICAM-1, and diminished secretion of pro-inflammatory cytokines [35].

In the present study, we used IVM to demonstrate that PARP inactivation in leukocytes drastically attenuated leukocyte adhesion/migration to and across cortical vessels in two in vivo models, LPS-induced systemic inflammation, and TNFα-induced localized encephalitis (Fig. 2), whereas autologous leukocytes displayed regular adhesion. PARPko animals exhibited attenuated inflammation-caused permeability vs. WT mice (Fig. 1c). PARP absence resulted in a decreased number of adherent leukocytes when injected into WT mice or an enhanced number of adherent leukocytes when WT leukocytes were introduced into PARPko mice (Fig. 3). Interestingly, the cells from the PARPko mice were able to adhere much better in the WT mice vs. the PARPko mirroring results in Fig. 1c, whereas leukocyte adhesion in the PARPko animals was reduced too, not only the leukocyte adhesion machinery was affected in these animals but also the BBB was less impaired [35]. Our in vivo results were reproduced in vitro where PARPi-treated monocytes showed substantially reduced adhesion to and migration across BMVEC monolayers (Fig. 4). The present study determined that inactivation of PARP in primary human monocytes tempered barrier dysfunction (denoted by a TEER reduction) (Fig. 4), with diminished monocyte engagement of the brain endothelium (adhesion/migration).

We have explored the idea that PARP inactivation results in conformational changes of active integrin and/or total integrin expression. Inactivation of PARP and Rac1 inhibition attenuated active integrin β1 expression (Fig. 5), while expression of total VLA-4 was unchanged, demonstrating the importance of VLA-4 conformation. Conformational activation of integrin apparently exposes the VCAM-1 binding site [53], facilitating both tethering and rolling [54]. It has been shown that memory T cells, constitutively expressing activation/ligand-induced epitopes on β1 integrins, exhibit significantly higher rates of attachment and accumulation on VCAM-1 expressing cells compared to other T cell subsets without active epitope expression [9, 55]. Previously, we [8, 9] and others [56] have shown a link between conformational VLA-4 activation and Rac1 pathways (inhibition of GSK3β or Rac-1, or CB2 activation), leading to lessened expression of the active VLA-4 form resulted in attenuated monocyte adhesion/migration in BMVEC [8, 9].

LFA-1 inside-out activation was stimulated by PMA in primary monocytes, and Rac1 inhibition or PARP inactivation decreased LFA-1 conformational change, whereas RhoA inhibition had no effect (Fig. 6). It has been shown that Rac1 plays an important role in LFA-1 activation in T cells [57]. Mutations of the LFA-1 β2-subunit prevented LFA-1 surface expression [58], resulting in type I leukocyte adhesion deficiency, which is induced by small Rho GTPase signaling [59].

Small GTPases (Rho, Rac, and Cdc42) play a vital role in transendothelial leukocyte migration, oxidative stress, and inflammation by linking surface receptors and the actin cytoskeleton [60, 61]. Rac1 and Cdc42 in leukocytes control lamellipodia formation, cell polarity, and direct migration, while RhoA controls leukocyte tail retraction during transmigration [62]. Given outcomes of functional assays, we measured RhoA and Rac1 activation after MCP-1 or PMA stimulation of monocytes and showed a decrease in active GTPases after PARP inactivation, resulting in diminution of actin cytoskeletal rearrangements (Fig. 7), which play a central role in cell migration [8, 63–65]. F-actin-rich lamellipodia formation caused by stimulating actin filament disassembly near the pointed ends supplies actin monomers for polymerization [66]. MCP-1 stimulation resulted in amplified actin rearrangements in monocytes (high F/G actin ratio), whereas PARP inactivation substantially diminished F/G actin ratio (Fig. 7). RhoA and Rac1 inhibitors exhibited analogous results, further signifying their involvement in this process.

There is a good amount of literature suggesting that PARP serves as a key component in nuclear factor kappa B, NFkB, activation, and consequently to transcription and expression of many inflammatory genes [35, 39, 67–69]. In brain-resident microglia, TNFα activates PARP in a DNA damage-independent manner, involving phosphatidyl choline-specific phospholipase and the MAPK kinase, ERK [70].

Rho GTPases are known to participate in MAPK kinase signaling activation [71]. In hepatocytes, MAPK kinase downregulation significantly abrogated the Rho pathway and cytoskeleton reorganization [72]. It is widely accepted that there is a relationship between PARP and MAPK, suggesting that they might stimulate each other in a feedback cycle [73]. Both MAPK kinases and Rho GTPases are involved in cytoskeleton rearrangements and cell migration [9, 71, 74]. Deeper understanding of these signal transduction interrelations may contribute to the development of more fine-tuned anti-inflammatory therapeutics.

Oxidative stress plays an important role in many neuroinflammatory conditions [75, 76]. Prolonged oxidative stress might lead to alterations in actin cytoskeleton in leukocytes [77]. In recent years, numerous PARP functions have been discovered, involving the regulation of oxidative stress and inflammation via complex interlinked signaling networks [73, 78]. Free radicals are potent activators of PARP; consequently, augmented PARP activity is usually associated with oxidative/nitrative stress [79]. Although the latest reports have implicated a bidirectional relationship, PARP inhibition itself is capable of reducing ROS formation in various pathologies [51, 79, 80]. PARP activation affects the energy balance of cells by NAD^+^ depletion, thereby causing compromised glycolysis and ATP depletion [78]. Functional behavior of immune cells (macrophages) is intensely coordinated by actin cytoskeleton rearrangements, a process in which ATP and NAD^+^ also play an indispensable role [81]. In this report, we did not study PARP activation-stimulated energy depletion and its effects on leukocyte function. However, understanding the role of PARP-stimulated NAD^+^ depletion and leukocyte actin cytoskeleton machinery during inflammation is required in the future. Two PARPi that were used in this study, talazoparib and olaparib, are now in clinical trials for treatment of several cancers (phase II and III) [10], promising speedy translation in evolving therapeutic route in various inflammatory diseases [82]. While the anti-inflammatory effects of PARP inhibition are gaining momentum [83], exact mechanisms are still largely unknown (especially in the CNS).

The concept that BBB damage is tied to leukocyte migration into the CNS is well accepted [28]. Therefore, attenuated adhesion of inflammatory cells to the brain endothelium via inhibition of PARP may prove valuable in stroke, MS or infection-associated encephalitis, and other inflammatory disorders. Particularly for the field of stroke, the inflammatory response has Janus-faced effects. Inflammation is known to have deleterious effects [32, 84–86], but is also known to initiate regenerative processes, leading to the perspective that inflammation is a necessary part of the physiological reaction towards tissue repair and plasticity [32–34]. In this perspective, effects resulting in BBB opening after an acute stimulus (during ischemia/reperfusion) and PARP inhibition might be very valuable, but long-term effects of PARP inhibition need to be investigated further before such an intervention could be considered clearly beneficial. Here, we have described previously unrecognized effects of PARP inhibition on diminution of VLA-4 and LFA-1 activation, mitigation of actin cytoskeleton rearrangements via small GTPases, and attenuation of leukocyte adhesion and migration across the BBB. Attenuation of leukocyte invasion is associated with minimized brain damage and might serve to translate these experimental findings into clinical therapies.

## Conclusions

Neuroinflammation is accompanied by leukocyte infiltration into the brain resulting in subsequent BBB dysfunction and significantly contributing to morbidity in ischemic stroke, multiple sclerosis, encephalitis, and traumatic brain injury. Pathways that reduce the inflammatory potential of leukocytes would prevent such damage. Our results suggest PARP inhibition in leukocytes as a novel approach to BBB protection in the setting of endothelial dysfunction caused by inflammation-induced leukocyte engagement.

## Additional file

Additional file 1: Figure S1. PARPi decrease PARP activity in a dose-dependent manner. PARP activity of primary monocytes treated with different concentrations of PARPi AIQ (A) and olaparib (B). Results are presented as the mean ± SEM (**P* < 0.05, ***P* < 0.01 vs. untreated control) from two independent experiments for at least three replicates. Figure S2. PARP activity significantly diminished in ex vivo treated mouse leukocytes. (A) PARP activity was measured in freshly isolated and ex vivo PARPi-treated mouse leukocytes. Data are presented as mean ± SEM for at least three replicates from three/four donor mice. ****P* < 0.005 indicate significance vs. non-treated. (B) Flow cytometry data presenting leukocyte profile of isolated leukocytes with or without PARPi treatment.

## Abbreviations

AIQ-5: Aminoisoquinolinone
BBB: Blood-brain barrier
BMVEC: Brain microvascular endothelial cells
EB47: 5′-Deoxy-5′-[4-[2-[(2, 3-dihydro-1-oxo-1H-isoindol-4-yl) amino]-2-oxoethyl]-1-piperazinyl]-5′-oxoadenosine dihydrochloride
FACS: Fluorescence-activated cell sorting
IC: Intracerebral
ICAM-1: Intercellular adhesion molecule 1
IVM: Intravital videomicroscopy
LPS: Lipopolysaccharide
MCP-1: Monocyte chemotactic protein-1
MFI: Mean fluorescence intensity
MS: Multiple sclerosis
MS: Multiple sclerosis
Na-F: Sodium fluorescein
NFkB: Nuclear factor kappa B
PARP: Poly(ADP-ribose) polymerase
PARPi: Poly(ADP-ribose) polymerase inhibitors
TBI: Traumatic brain injury
TEER: Transendothelial electrical resistance
VCAM-1: Vascular cell adhesion molecule 1
