# Supplementary material for: PARP inhibition in leukocytes diminishes inflammation via effects on integrins/cytoskeleton and protects the blood-brain barrier
Source: J Neuroinflammation. 2016 Sep 27;13:254. doi: 10.1186/s12974-016-0729-x (PMC5039899; doi:10.1186/s12974-016-0729-x)
Supplement: Additional file 1: Figure S1. — PARPi decrease PARP activity in a dose-dependent manner. PARP activity of primary monocytes treated with different concentrations of PARPi AIQ (A) and olaparib (B). Results are presented as the mean ± SEM (*P < 0.05, **P < 0.01 vs. untreated control) from two independent experiments for at least three replicates. Figure S2. PARP activity significantly diminished in ex vivo treated mouse leukocytes. (A) PARP activity was measured in freshly isolated and ex vivo PARPi-treated mouse leukocytes. Data are presented as mean ± SEM for at least three replicates from three/four donor mice. ***P < 0.005 indicate significance vs. non-treated. (B) Flow cytometry data presenting leukocyte profile of isolated leukocytes with or without PARPi treatment. [file 12974_2016_729_MOESM1_ESM.docx]

**Additional Figures for manuscript titled “PARP inhibition in leukocytes diminishes inflammation via effects on integrins/cytoskeleton and protects the blood brain barrier”**

Slava Rom^1,2^, Viviana Zuluaga-Ramirez^1^, Nancy L. Reichenbach^1^, Holly Dykstra^1^, Sachin Gajghate^1^, Pal Pacher^3^ and Yuri Persidsky^1,2^


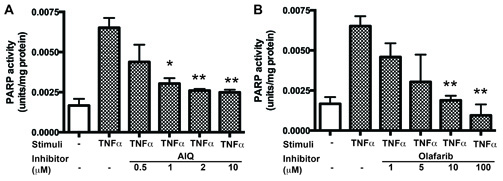


Additional Figure 1. *PARPi decrease PARP activity in a dose-dependents manner.* PARP activity of primary monocytes treated with different concentrations of PARPi AIQ (A) and Olaparib (B). Results are presented as the mean ±SEM (*P<0.05, **P<0.01 vs. untreated control) from two independent experiments for at least three replicates.


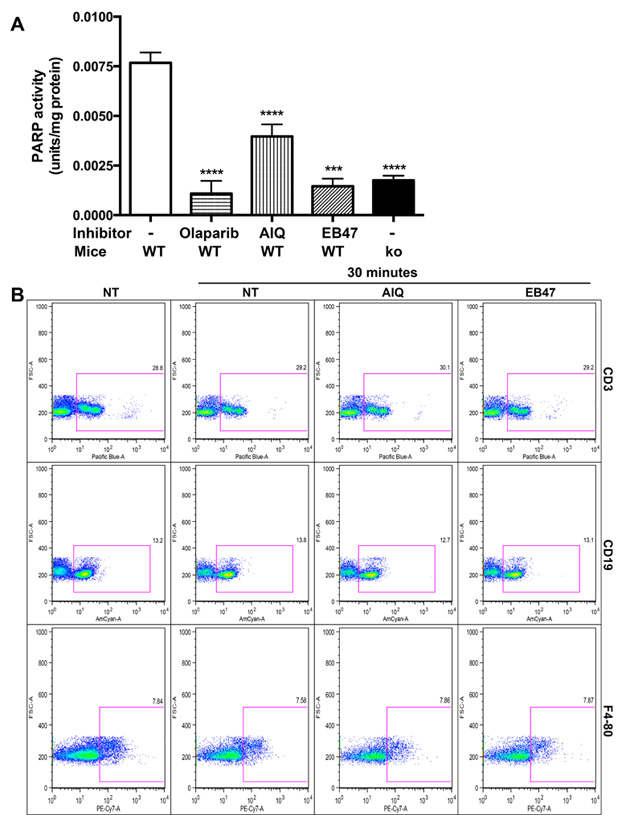
 Additional Figure 2. *PARP activity significantly diminished in ex vivo treated mouse leukocytes.* (A) PARP activity was measured in freshly isolated and *ex vivo* PARPi treated mouse leukocytes. Data are presented as mean ±SEM for at least three replicates from three/four donor mice. ***P<0.005 indicate significance vs. non-treated. (B) Flow cytometry data presenting leukocyte profile of isolated leukocytes with or without PARPi treatment.
